# A HOUSING COALITION IN DETROIT MICHIGAN PLANS AHEAD FOR INDIVIDUAL AND COMMUNITY-LEVEL EMERGENCIES

**DOI:** 10.1093/geroni/igae098.0294

**Published:** 2024-12-31

**Authors:** Tam Perry, Zach Kilgore, Michele Watkins, Claudia Sanford, Brenda Price, Dennis Archambault

**Affiliations:** Wayne State University, Detroit, Michigan, United States; CSI Support & Development, Warren, Michigan, United States; Volunteers of America Michigan, Southfield, Michigan, United States; United Community Housing Coalition, Detroit, Michigan, United States; AARP Michigan, Lansing, Minnesota, United States; Authority Health, Detroit, Michigan, United States

## Abstract

Since its founding in 2013, Senior Housing Preservation-Detroit has worked tirelessly to address the displacement of older adults, the loss of senior housing as the city gentrifies; and ways to improve quality of life to those living in senior housing. As we turn the corner on the acute phase of the COVID-19 pandemic, the members of Senior Housing Preservation-Detroit are compelled to take stock of the lessons learned from the urgent, emergency context of serving older adults in the city of Detroit, and particularly those living in senior buildings. Whether the next emergency is isolated to a single building (in case of power outages or flooding) or affects many buildings (due to another infectious disease, our changing climate, etc.), we know that preparation must continue to happen on an individual and building level. Building staff and residents alike must be ready to respond to multi-faceted problems, and there must be better plans for serving a diverse senior population. SHP-D has explored ways to reduce risks to seniors in the next emergency, including improving communication, implementing preparedness plans, and enhancing the physical resiliency of buildings. This presentation will focus on the coalition’s work on emergency planning to share knowledge which may benefit other communities planning for older adults in emergencies.

